# Identifying associations between health services operational factors and health experience for patients with type 2 diabetes in Iran

**DOI:** 10.1186/s12913-021-06932-0

**Published:** 2021-08-31

**Authors:** Mahdi Mahdavi, Mahboubeh Parsaeian, Shiva Borzouei, Reza Majdzadeh

**Affiliations:** 1 The Bernard Lown Scholar in Cardiovascular Health, Harvard T.H. Chan School of Public Health, Boston, USA; 2 National Institute for Health Research, Tehran University of Medical Sciences, Postal address: No 70, Bozorgmehr st, Tehran, Iran; 3 School of Public Health, Tehran University of Medical Sciences, Tehran, Iran; 4 Department of Internal Medicine, School of Medicine, Hamadan University of Medical Sciences, Hamedan, Iran; 5 Knowledge Utilization Research Center and Community-Based Participatory-Research Center, Tehran University of Medical Sciences, Tehran, Iran

**Keywords:** Type 2 diabetes, Quality of life, Satisfaction with healthcare, Satisfaction with health status, Patient experience, Healthcare structure, Healthcare process, Health services operations

## Abstract

**Background:**

Facing limited health resources, healthcare providers need to rely on health service delivery models that produce the best clinical outcomes and patient experience. We aimed to contribute to developing a patient experience-based type 2 diabetes service delivery model by identifying operational structures and processes of care that were associated with clinical outcome, health experience, and service experience.

**Methods:**

We conducted a cross-sectional survey of type 2 diabetes patients between January 2019 to February 2020. Having adjusted for demand variables, we examined relationships between independent variables (behaviours, services/processes, and structures) and three categories of dependent variables; clinical outcomes (HbA1c and fasting blood glucose), health experience (EuroQol quality of life (EQ-5D), evaluation of quality of life (visual analgene scale of EQ-5D), and satisfaction with overall health status), and service experience (evaluation of diabetes services in comparison with worst and best imaginable diabetes services and satisfaction with diabetes services). We analysed data using multivariate linear regression models using Stata software.

**Results:**

After adjusting for demand variables; structures, diabetes-specific health behaviours, and processes explained up to 22, 12, and 9% of the variance in the outcomes, respectively. Based on significant associations between the diabetes service operations and outcomes, the components of an experience-based service delivery model included the structural elements (continuity of care, redistribution of task to low-cost resources, and improved access to provider), behaviours (improved patient awareness and adherence), and process elements (reduced variation in service utilization, increased responsiveness, caring, comprehensiveness of care, and shared decision-making).

**Conclusions:**

Based on the extent of explained variance and identified significant variables, health services operational factors that determine patient-reported outcomes for patients with type 2 diabetes in Iran were identified, which focus on improving continuity of care and access to providers at the first place, improving adherence to care at the second, and various operational process variables at the third place.

**Supplementary Information:**

The online version contains supplementary material available at 10.1186/s12913-021-06932-0.

## Introduction

Diabetes is amongst the leading causes of morbidities and mortality around the world. The prevalence of diabetes amounted to 451 million in 2017 globally, of which 90% were Type 2 Diabetes (T2D). The number of adults aged 18 years and older living with diabetes is expected to increase to 693 million by 2045 [1]. The proportion of Iranian adults aged 25–70 years who were living with diabetes mellitus (DM) was 11.4% between 2007 and 2011 [2]. It is estimated that 85.5% of patients with DM have T2D in Iran [3].

A review study showed that the Iranian healthcare delivery system is struggling to improve the operations management of health services for patients with T2D [4]. Two major shortcomings hinder improving operations management of T2D services. Firstly, most evaluation studies examine single dimensions of T2D services in Iran while they report deficits in the operations management of health services for patients with T2D in terms of access to care, inefficient use of human resources, disorganized care processes, lack of continuity of care, and so forth [5, 6]. However, no study evaluates an operational model of T2D services. We define an operational model as health services activities that use health structures and resources to meet patient demands for better health outcomes [7, 8]. Therefore, there is an urgent need for research that examines multiple dimensions of services as included in operational models of health services.

Secondly, since there is no permanent cure for patients with T2D, it is of prominent importance that health services for this group of patients effectively maintain clinical states and quality of life and pay attention to patient experience with services [9]. In this vein, health authorities call for patient-centred care (PCC) models [10] that aspire to build healthcare operations around patient needs, preferences, and expectations [11]. Yet, as a widely known phenomenon, service delivery models are driven by administrators and medical professionals in a top-down fashion without engaging patients in designing care processes. A review study showed that PCC missions are not fully accomplished even in developed countries [12]. In Iran as a developing country, due to the lack of holistic understanding of patient needs and preferences, patient experience with care remains completely untouched.

We aimed to examine relationships between operational factors and health experience and service experience of the patients with T2D in Iran. This research identifies the elements of healthcare operational structures and processes that are associated with patient report outcomes in terms of clinical outcomes (HbA1c and fasting blood glucose), health states (EuroQol quality of life (EQ-5D), evaluation of the quality of life (visual analgene scale of EQ-5D), satisfaction with health status, and service experience (evaluation of diabetes services and satisfaction with diabetes services). Our study allows an experience-based type 2 diabetes service delivery model to emerge from the empirical analysis of clinical outcomes and patient experience.

## Materials and methods

**Fig. 1:**
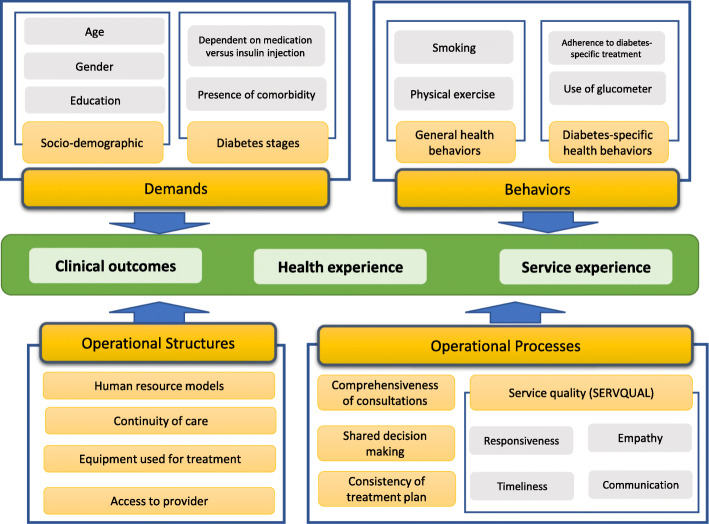
Conceptual framework: components of service delivery model for patients with type 2 diabetes (adopted from Mahdavi et al. [13])

Health services operational models provide methods to identify the elements of services activities that are associated with patient experience [14]. The health services operational model is defined as a simplified description of healthcare processes that use structures to improve patient outcomes and respond to patient demands [7]. The main elements of the operational model comprise demands, processes (service activities, service use, costs, and quality), structures (human resources, access, and equipment), and patient behaviours [8, 13] to improve outcomes and patient experience (Fig. 1).

### Study design, settings, and participants

We conducted a cross-sectional survey of health service operations and outcomes among patients with type 2 diabetes. We aimed to include samples from major provider organizations of diabetes care in the country, which consisted of the Ministry of Health and Medical Education (MoHME), Social Security Organization, other public sector organizations, and the for-profit private sector. We relied on a convenient sampling and included one clinic per provider. Three trained interviewers conducted data collection. Data collection was continuously performed to reach the target sample. In all data collection settings, it was completed in 3 months. We only included patients with T2D that were not complicated. Therefore, patients who regularly need to be hospitalized were excluded. Our methods were carried out following guidelines for reporting observational studies (Strengthening the reporting of observational studies in epidemiology (STROBE) statement) [15].

We used the sample size formula $n=\frac{{Z^2}_{1-\frac{\alpha\ }{2}}\times p\times \left(1-p\right)}{d^2}$ to determine the sample size [16]. To obtain a maximum sample size, we considered *p* = 0.5. The significance level and relative error were considered 0.05 and 10% (d = 0.1 × 0.5 = 0.05). With these assumptions, the total sample size was 400. After adjusting for 85% response rate, the total sample size was 472 participants. We divided this sample equally between four providers, ending up with 118 participants per provider.

### Variables

#### Demand variables

We described demands by demographic and socioeconomic indicators and diabetes stage (Table 1). Demographic and socioeconomic indicators consisted of age, gender, and education. Diabetes stages refer to two variables; whether the patient is dependent on medications or medications and/or insulin and whether a patient has at least one of diabetes comorbidities; a problem with heart, eyes, kidney, and feet and hypertension.

**Table 1:** Variables and measurement scales for demand and health behaviour of patients with type 2 diabetes

Components	Variables	Measurement scale ^a^
Demand	Age	Year (min: 22, max: 91)
	Sex	Female (0), male (1)
	Education	Some years of schooling (0), high school diploma (1), university education (2)
	Diabetes stage	Patient with T2D is dependent on medication (0) Patient with T2D is dependent on medication and/or insulin (1)
	Comorbidity	Patient with T2D has no comorbidity (0) Patient with T2D has at least one chronic comorbidity (1)
Generic health behaviours	Physical activities	Insufficient physical activity: physical activity < 500 Metabolic Equivalents per week (0) Sufficient physical activity: physical activity≥500 Metabolic Equivalents per week (1)
	Smoking	Non-smoker (0), former smoker (1), current smoker (2)
Diabetes-specific health behaviours	Adherence to treatment	Composite measure of adherence to treatment consisting of adherence to advice for diet, medication, and/or insulin injection in a Likert scale from 1 for a least adherence to 5 for complete adherence.
	Frequency of the use of glucometer by the patient	Several times per day (0), once per day (1), once per some days (2), once per some weeks (3), no use of glucometer (4)

#### Patient behaviours

Behaviour was defined as generic health behaviour and disease-specific behaviour (Table 1). The generic health behaviour comprised physical activity and smoking. Physical activity was defined in terms of metabolic equivalents (METs) with 500 METs (equates to 150 min of moderate or vigorous activity) per week as a cut-off point to determine if physical activity is sufficient [17]. Based on smoking, we categorized participants into three groups: current smokers, former smokers, or non-smokers. Diabetes-specific behaviours refer to patient adherence to diabetes-specific treatment and the use of glucometer by the diabetes patients. Diabetes-specific treatment regards treatment recommendations in terms of adherence to diet, taking medication, and insulin injection.

#### Outcomes

Outcomes measures consisted out of clinical outcomes, health experience, and service experience (Table 2). Clinical outcomes were measured through HbA1c (mmol/mol) and fasting blood glucose (FBS) level (mg/dl) [18]. In one of the studied clinics, these were taken from patient records. In three clinics, both outcomes were self-reported by patients. Health experience comprised of the perception of quality of life and two classes of health status evaluation (visual analgene scale (VAS) of quality of life and satisfaction with health status). Perception of quality of life was measured through EuroQol EQ-5D-5L in terms of five dimensions: mobility, self-care, usual activity, pain/discomfort, and anxiety/depression. On each dimension, valid responses had five options from no problem to severe problem. The individual’s utility score of EQ-5D was calculated from all five dimensions using the index developed for the Iranian population [19]. Satisfaction with health status, as the second class of health status evaluation, was measured through a single question in a Likert scale. Service experience comprised of an evaluation of diabetes services and satisfaction with diabetes services. The evaluation of diabetes services was conducted in comparison to the worst and best imaginable diabetes services. Satisfaction with services refers to satisfaction with whole diabetes services that patient receives from providers. It was measured through a single question on a Likert scale.

**Table 2:** Variables and measurement scales for the outcomes, operational structures, and operational processes of health services for patients with type 2 diabetes

Components	Variables	Definition	Measurement scale
Clinical outcomes	Glycated haemoglobin (HbA1c)	Glycated haemoglobin (HbA1c)	Mmol/mol
	Fasting blood glucose (FBS) level	Fasting blood glucose (FBS)	Mg/dl
Health experience	Perceived quality of life	Perception of quality of life was measured through EuroQol EQ-5D-5L.	Utility score of EQ-5D between 0 for death and 1 for full health.
	Evaluation of health status	Health status evaluation refers to comparing health status to the best and worst imaginable health status.	Visual analgene scale (VAS) of quality of life from 0 to 100 for death and full health.
	Satisfaction with health status	Satisfaction with health status refers to judgement made about overall health status based on the patient’s interval values.	Measured in Likert scale from completely dissatisfied to completely satisfied and then standardized between 0 and 100.
Service experience	Evaluation of health services	The evaluation of diabetes services refers to judgement made about the overall quality of diabetes services by comparing the services with the worst and best imaginable diabetes services.	Measured on a scale between 0 for the worst and 100 for the best diabetes services.
	Satisfaction with type 2 diabetes services	Satisfaction with services refers to a judgement made about overall diabetes service quality based on patient’s interval values.	Measured in a Likert scale from completely dissatisfied to completely satisfied. It was also standardized between 0 and 100.
Operational structures	Human resource models	A human resource model refers to the main type of healthcare providers that provide diabetes services. Human resource models are categorized based on the type of medical professionals e.g. nurse, GP, and specialized medical doctor. Resource models are developed by quantifying the number of visits and time spent by medical professionals for patient care.	Only family physician or general practitioner (0) Family physician or general practitioner & specialist physician (1) Only specialist physician (2)
	Access to diabetes services	This refers to perceived overall access to a care provider.	Likert scale from strongly disagree (1) to strongly agree (5).
	Continuity of care	This refers to whether patients have a regular medical professional such as GP or specialist (continuity) or patients are seen by a different provider for every visit (no continuity).	Being visited by the same doctor in every visit Being visited by a new doctor in every visit
	Equipment	This refers to if the equipment used to treat the patients are up to date and modern.	Likert scale from strongly disagree (1) to strongly agree (5).
Operational processes	Comprehensiveness of consultation	The comprehensiveness of consultation determines if all diabetes-related questions of the patient were answered during the consultation visit.	Likert scale from strongly disagree (1) to strongly agree (5).
	Shared decision making	Shared decision-making refers to involving patients in deciding for their care.	Likert scale from strongly disagree (1) to strongly agree (5).
	Consistency of treatment plans	Consistency of treatment plans refers to the situation that if providers involved in the care of the patient provided similar advice and recommendations.	Likert scale from strongly disagree (1) to strongly agree (5).
	Perceived service quality	Perceived service quality refers to five dimensions of short SERVQUAL comprising responsiveness of providers, timeliness, caring providers, politeness of providers, and communication between the patients and providers.	In each dimension, a Likert scale from strongly disagree (1) to strongly agree (5).

#### Structure of diabetes care

The structure contained four factors; human resource model, access to a provider, continuity of care, and the status of equipment used to treat diabetes patients [13]. The human resource model refers to the types of human resources that were employed to provide health services for patients with T2D (Table 2). Continuity of care was measured through a question ‘are you visited by a new doctor in every visit?’. If the patient answered ‘No’ to this question, diabetes care had continuity, otherwise, there was no continuity of care.

Processes of diabetes care.

Diabetes care processes were measured through the comprehensiveness of consultation, shared decision making, the consistency of treatment plans, responsiveness of providers, timeliness, caring providers, politeness of providers, and communication between the patients and providers (Table 2) [13]. Responsiveness of providers measured whether providers promptly respond to patient demands. Timeliness measured the degree to which a provider delivered services in the planned time.

### Instrument for data collection

The data collection instrument used in this research was previously administered to 1459 patients with T2D in six European countries to study diabetes service operations of the regional healthcare provider networks [13, 20, 21]. After translation, the questionnaire was checked for validity by a group of experts specialized in internal medicine and diabetes treatment. The questionnaire was adapted to the local condition of study settings. The questionnaire included multiple instruments including the EuroQol EQ-5D quality of life instrument that has been validated for Farsi-speaking individuals [22]. We did not, therefore, check the reliability of the EQ-5D instrument in this research. The internal consistency of process variables was checked through Cronbach’s alpha. It ranged from 0.92 for ‘responsiveness of providers’ to 0.94 for ‘comprehensiveness of medical consultation’.

### Analysis methods

We examined associations between the outcomes and operational variables using multivariate linear regression models for continuous outcomes. We did not build a single construct for outcome by combining all outcomes. Per outcome, we developed six regression models to determine variables that explain the outcome. All analyses of outcomes were integrated into the Results and Discussion sections to allow developing an experience-based service delivery model.

The demographic factors were controlled in the first model. The second model controlled the main effect of the variables for demographic factors and diabetes stages. The third model included demographic factors, diabetes stages, and general health behaviours. The fourth model had the variables of the third model and added the diabetes-specific behaviours to the regression model. The fifth model had the variables of the fourth model and added variables for the structure of diabetes care to the regression model. And the sixth model contained the variables of the fifth model and added the variables of care processes. R^2^ and change in R^2^ from one model to the next one were used to determine the contribution of each component of the operational model to clinical outcomes and the patient experience measures. To identify variables of the subcomponents that constitute our service delivery model, we reported the sixth regression model of all seven outcomes. We reported beta coefficients (β) and *P* values in the manuscript and reported more details of regression analyses including beta coefficients, confidence interval of beta coefficients, and *p* values in Additional file 1. All analyses were conducted using Stata version 14.1.

## Results

Data were collected from healthcare settings that are located in the urban areas and treat T2D patients. Overall, 521 questionnaires were administered. With 94% response rate, diabetes patients returned 492 questionnaires of which 486 questionnaires were used in the analysis.

The age group 60–69 years was the largest group with 37.8% of the study sample. In the next rank, the age group 50–59 years comprised 34.4% of the study sample (Table 3). A large percentage of participants were females. Only a small percentage of participants (11.3%) had a university education. Most participants were dependent on medications (64.6%). Regarding comorbidities, 42.6% of participants reported no comorbidity.

**Table 3:** Descriptive characteristics of study participants

Variable		n	%
Age	30-49 year	55	11.5
	50–59 year	165	34.4
	60–69 year	181	37.8
	≥70 year	78	16.3
Sex	Male	340	70.7
	Female	141	29.3
Education	Some high school	300	62.8
	Completed high school/diploma	124	25.9
	University education	54	11.3
Stage of diabetes	Stage of type 2 diabetes treated by diet & medication	306	64.6
	Stage of type 2 diabetes treated by diet & medication & insulin injection	168	35.4
Comorbidity	No comorbidity	206	42.6
	At least one comorbidity	278	57.4

### Model predictions

The contributions of model components to the seven outcomes are given in Table 4. Age, gender, and education altogether explained the largest percentage of variation in the quality of life. The diabetes stage, with two variables dependency on medication or insulin injection and comorbidity, had the largest contribution to clinical outcomes HbA1c level. General health behaviours comprise much of explained variance in the HbA1c level. Diabetes-specific health behaviour explained up to 12% of the variance in the outcomes with the largest share for satisfaction with health services. The structure of diabetes care explained 3–22% of the variance in the outcomes. It has the largest contribution to explaining service experience. The process of diabetes care explained up to 9% of the variance in the outcomes with the largest contribution to the evaluation of diabetes services.

**Table 4:** The extent of explained variance in outcomes of type 2 diabetes services

	Statistical	Demographic factors (1)	1+ Diabetes stages (2)	2 + General health behaviours (3)	3 + Diabetes-specific health behaviours (4)	4 + Diabetes care structure (5)	5 + Diabetes care processes
HbA1c level	R square	2%	12%	18%	20%	25%	30%
	p-value	0.17	*p* < .001	p < .001	p < .001	p < .001	*p* < .001
	frequency	254	249	247	232	221	212
Fasting Blood Sugar	R square	0	13%	18%	21%	25%	23%
	p-value of F test	0.32	p < .001	p < .001	*p* < .001	p < .001	p < .001
	number	439	249	247	232	221	352
EuroQol EQ-5D quality of life	R square	12%	18%	21%	23%	28%	29%
	p-value of f test	p < .001	p < .001	p < .001	p < .001	p < .001	p < .001
	number	466	455	449	410	386	371
Visual analogue scale of EQ-5D	R square	6%	8%	13%	18%	21%	26%
	p-value of f test	p < .001	p < .001	p < .001	p < .001	p < .001	p < .001
	number	459	448	443	404	382	369
Satisfaction with health status	R square	4%	7%	8%	13%	17%	18%
	p-value of f test	0.002	p < .001	p < .001	p < .001	p < .001	p < .001
	number	466	455	449	409	384	384
Satisfaction with health services	R square	1%	2%	4%	16%	38%	44%
	p-value of F test	0.51	0.27	0.06	p < .001	p < .001	p < .001
	number	457	449	443	403	380	365
Evaluation of health services in comparison with best imaginable services	R square	1%	3%	5%	14%	36%	45%
	p-value of F test	0.16	0.02	0.003	p < .001	p < .001	p < .001
	number	455	447	442	402	380	365

The final regression models, which include all components demand, behaviour, structure, and process, explained 18 to 45% of the variance in the outcomes; the lowest extent of explained outcome with 18% belongs to satisfaction with health status and the largest with 45% belongs to the evaluation of diabetes services in comparison with best and worst imaginable services. Satisfaction with diabetes services with 44% was the second outcome with the highest extent of explained variance.

### Diabetes stages

The analyses of relationships between the components of the operational model and the outcomes are given in Table 5. An online appendix provides detailed information including predictor variables, beta coefficient, confidence interval of coefficients, and *p*-value for health outcomes analysed. Diabetes stage variables were associated with the clinical outcomes. Being dependent on medication and/or insulin was associated with increased HbA1c (β = 1.33, *p* < .05) as well as increased FBS level (β = 30.67, *p* < .05). Another variable of diabetes state, comorbidity was associated with worsening outcomes; it was significantly associated with increased FBS level (β = 1.33, *p* < .05) and decreases in three other health-related outcomes; quality of life (β = − 0.05, *p* < .05), the evaluation of quality of life (β = − 5.25, p < .05), and satisfaction with health status (β = − 0.34, *p* < .05).

**Table 5:** Regression analysis of relationships between the outcomes and demographic, socioeconomic, health behavior, and operational factors among patients with type 2 diabetes ^a^

	Variable	HbA1c level	Fasting Blood Sugar	EuroQol EQ-5D quality of life	Visual analogue scale of EQ-5D	Satisfaction with health status	Satisfaction with health services	Evaluation of health services
Demographic and socio-economic factors	Age	0.01	−0.24	−0.00	−0.13	0.23	−0.19	0.06
	Sex							
	Female (reference)	1		1		1	1	
	Male	−0.26	−10.61	0.08*	5.53*	5.64	1.06	1.64
	Education							
	Some years of schooling	1				1	1	
	High school diploma	0.46	−6.13	−0.01	4.11	4.78	−3.07	− 0.50
	University education	0.42	8.19	0	6.50	1.86	−3.24	−1.66
Diabetes stages	Disease state							
	Dependent on medication (ref)	1	1	1	1	1	1	1
	Medication and/or insulin-dependent	1.33*	30.67*	−0.03	−2.67	−3.01	−1.83	−0.25
	Chronic comorbidity							
	Having no other chronic comorbidity (ref)	1	1	1	1	1	1	1
	Having at least one other chronic comorbidity	−0.23	13.87*	−0.05*	−5.25*	−8.42*	0.37	−1.06
General health behaviours	Physical exercises							
	Physical exercises < 500 Metabolic Equivalents per week	1	1	1	1	1	1	1
	Physical exercises ≥500 Metabolic Equivalents per week	−0.07	−17.71*	0.02	11.33*	7.23*	−0.04	−1.38
	Smoking							
	Non-smoker	1				1	1	1
	Former smoker	0.23	−0.29	− 0.09*	− 0.33	2.35	−0.61	5.31
	Current smoker	1.58*	13.14	− 0.05	−4.28	−3.08*	−8.98	−6.04
Diabetes-specific health behaviours	Adherence to treatment (diet, medication, and/or insulin injection)	−0.33*	−10.03*	0.02*	3.64*	3.63*	3.31*	2.81*
	Use of glucometer							
	Several times per day	1	1	1	1	1	1	1
	Once per day	1.23	−7.29	0.02	−4.10	−8.58	1.78	5.25
	Once per some days	1.46*	16.34	0.03	−7.93	−8.46	4.34	4.34
	Once per some weeks	1.12*	9.94	0.02	−4.18	−3.28	3.92	6.10
	No use of glucometer	1.2	7.40	0.03	−10.72*	−10.87	3.33	4.20
Diabetes care structures	Human resource model							
	Only family physician or general practitioner	1	1	1	1	1	1	1
	Family physician or general practitioner & specialist physician	0.92	12.21	−0.01	−0.23	−8.05	5.59	2.62
	Only specialist physician	0.5	3.18	− 0.02	−2.76	−9.03	3.54	3.28
	Access to diabetes services	−0.09	−5.40	0.02*	0.64	1.42	5.50*	1.65
	Continuity of care							
	Being visited by a same doctor in every visit	1	1	1	1	1	1	1
	Being visited by new doctor in every visit	0.87*	28.21*	−0.01	−1.00	0.14	0.19	−3.73
	Up to date equipment for diabetes care	0.60*	16.18*	−0.02	− 3.45	−2.72	3.18	3.02
Diabetes care processes	Comprehensiveness of medical consultation	−0.51	−4.28	0.01	2.58	1.88	4.83*	1.95
	Patient involvement in care decision	0.77*	1.53	0	0.38	−1.62	−2.13	2.27
	Consistency of treatment medical plans and advises	−0.2	2.25	0.01	3.14*	1.90	−1.08	1.17
	Responsiveness of providers	−0.06	−4.71	0	2.75	3.89	3.96*	3.94*
	Timeliness of provider	0.08	7.59	−0.01	−1.45	−0.56	− 1.38	1.53
	Caring provider	0.27	−16.67*	0	−1.57	0.28	6.61*	6.59*
	Politeness of care provider	−0.15	1.63	0.02	6.59*	0.43	1.00	0.44
	Communication between patient and provider	−0.27	4.82	−0.01	−4.90*	−1.01	−2.72	−3.80

Notes: ^a^ Unstandardized coefficients (R2) of variables in relationship with type 2 diabetes care outcomes

* *p*-value<.05

### Health behaviours

Health behaviours, both general and diabetes-specific behaviours, were associated with the outcomes. Physical activity was associated with decreased FBS level (β = − 17.71, *p* < .05) and improved evaluation of health status (β = 11.33, p < .05) and satisfaction with health (β = 0.29, p < .05). Smoking was also associated with HbA1c level, quality of life, and satisfaction with health services. Current smoking was associated with increased level of HbA1c (β = 1.58, p < .05).

Adherence to treatment was significantly associated with all outcomes; it was associated with decreased level of HbA1c (β = − 0.33, *p* < .05) and FBS level (β = − 10.03, p < .05). On the other hand, it was significantly and directly associated with quality of life (β = 0.02, *p* < .05), evaluation of quality of life (β = 3.64, p < .05), satisfaction with health, satisfaction with health services, and evaluation of health services.

A decrease in the number of uses of glucometer was associated with an increased level of HbA1c and decreased level of evaluation of health status after controlling for dependency on insulin. The use of glucometer as ‘once per a couple of days’ and ‘once per a couple of weeks’ were associated with an increased level of HbA1c (β = 1.46 and β = 1.12, p < .05). Patients with no use of glucometer had 11% lower level of the evaluation of health status (β = − 10.72, *p* < .05) compared with patients who use glucometer several times per day.

### Structures of care

The variables of care structure included human resource models, access to a provider, continuity of care, and the state of equipment. Three models of human resource emerged from the analysis: ‘diabetes care by family physician or general practitioner’, ‘diabetes care by family physician or general practitioner & specialist physician’, and ‘diabetes care by specialist physician only’. No difference was observed between the three models of human resources in the analysis of all outcomes. Improved perceived access to services was associated with EQ-5D utility score (β = 0.02, *p* < .05) and satisfaction with health services (β = 5.52, p < .05). Continuity of care increased HbA1c level (β = 0.87, p < .05) and FBS level (β = 28.2, p < .05). More up-to-date equipment for diabetes care was associated with increased HbA1c and FBS levels.

### Diabetes care processes

Regression analyses showed an insignificant relationship between the utilization of routine GP visits, consultation visit by specialized internal medicine doctors, and endocrinologists and health outcomes. The only exception was the relationships between EQ-5D utility score and utilization of endocrinology visit, where by increasing in the number of visits, the utility score decreases (β = − 0.02, *p* < .01). Furthermore, total hours of care (sum of service time spent in all diabetes services) per patient per year was negatively associated with the EQ-5D utility score.

Concerning adherence to diabetes care standards, 37% of patients with GPs as their main caregiver, reported less than four visits per year, only 8% reported four visits per year, and 55% reported more than four visits per year. Of patients who reported medical specialists as their main care giver, 42% reported less than four visits per year, 30% of patients reported just four visits per year, and 28% had more than four visits per year.

Two dimensions of short SERVQUAL instrument ‘responsiveness’ and ‘caring’ were significantly associated with both satisfaction with health services and evaluation of diabetes services. One unit increase in responsiveness was associated with a 4% increase in service satisfaction (β = 3.96, *p* < .05) and evaluation of diabetes services (β = 3.94, p < .05). One unit of caring, measured alike responsiveness, increased service satisfaction and evaluation of diabetes services by 7% (β = 6.61 and β = 6.59 respectively, p < .05). Furthermore, caring was associated with FBS level.

## Discussion

Our empirical analyses identified variables of behaviours, operational structures, and operational processes that were significantly associated with clinical outcome, health experience, and service experience. These variables could be the ingredients of an experience-based type 2 diabetes service delivery model. The empirical analyses showed the total contribution of main components to the outcomes. In comparison between the main components, the structure of care explained a larger extent of variance in the outcomes than other components. The next rank was the diabetes-specific health behaviours with up to 12% of explained variation in the outcomes. The third rank belongs to the process of care with a maximum of 9% of explained variation in the outcomes.

Given the larger contribution of operational structures to the outcomes, we firstly discuss structure variables. Despite the variation in the level of expertise and the tariff schedule for medical professionals, no health outcome varied between the three models of human resources. An insignificant association between human resource models and the outcomes supports the use of less costly resource model i.e., GPs and family doctors instead of more expensive specialized doctors. These insignificant differences between human resources models also shed light on the efficiency of task shifting from specialized doctors towards GPs or family doctors [23–25]. Previous studies confirm this finding that patient-reported outcomes were similar between T2D services that were provided by GPs compared with services provided by specialists [26]. At the regional provider network level, research showed that with a larger role for diabetes GPs, the percentage of good-control diabetes patients increases [13].

Perceived access to the provider was also associated with the quality of life and service satisfaction, which confirms already established evidence on the importance of access to the provider for patient-centred care models [27]. The negative association between being treated by a new doctor in every visit and HbA1c and FBS level supports evidence that continuity of care positively affects health outcomes [28].

As far as the efficiency of services is concerned, the number of visits needs to be standardized which is also recommended by diabetes guidelines. We found a large variation in the number of routine visits per patient/year, showing overuse and underuse of routine GP or medical specialist visits at the same time. This indicates that care processes are not under control in the studied settings [29]. The average number of routine visits that patients received was higher than the 4 visits recommended by clinical guidelines [30]. The inefficiency of diabetes care is evident given the presence of 28–55% overuse of routine visits by the patients. There was a negative relationship between the number of visits and quality of life, which essentially implies reverse causality that by the decreased quality of life, the use of services increases [13].

The elements of diabetes care processes were mostly associated with service satisfaction and evaluation of diabetes services satisfaction. The comprehensiveness of care increases service satisfaction. Two dimensions of short SERVQUAL instrument ‘responsiveness’ and ‘caring’ were associated with service satisfaction and evaluation of diabetes services. Other dimensions of diabetes service quality were not associated with the outcomes. Caring was also associated with FBS level. Other studies support the association between these dimensions of short SERVQUAL instrument and satisfaction with services [13].

As predicting factors per outcome were identified in this research, specific interventions to change outcomes can be drawn. Structural components that are significantly associated with the outcomes comprise increased continuity of care, redistribution of tasks from internal medicine doctors to GPs or family doctors, and improved access in terms of facilitated appointment scheduling and/or shorter travelling time and/or waiting time in provider offices. Furthermore, facilitating access to self-test at home, such as test kits is a structural component. Process components comprise a reduction in the variance of service utilization through the standardization of routine visits [31] and tackling unnecessary overuse of services. Process indicators also include improving the responsiveness and caring behaviour of providers. In the next rank, we regard improving comprehensiveness of care and patient involvement in decision making as the subcomponents of care process. Our findings support certain elements of PCC models such as engagement, shared decision-making, holistic focus [11], emotional support, access to care, and continuity of care [32–34].

We controlled demographic and health state variables in our multivariate analysis. Age, sex, and education level were associated with quality of life. The stage of diabetes and comorbidity of chronic diseases were associated with health outcomes. Other studies also confirmed that comorbidities and stage of diabetes strongly influence patient outcomes [35]. Our findings showed that comorbidity of other chronic diseases had no association with HbA1c level but had significant association with the quality of life. This implies that even with comorbidities of diabetes, patients can be in good-control diabetes stage.

This study faced several limitations. The study design relied on cross-sectional surveys to measure outcomes and operational variables. The survey design, as being non-experimental, does not allow discovering causal relationships. We used a non-probabilistic sampling framework, given the limitation of research resources. Given this, study findings may not be generalized to larger populations of diabetes patients or provider settings. However, we may argue that we investigated typical cases with inclusion criteria that preclude major biases. The measurement of clinical outcomes was a concern in this research. We, therefore, used two clinical outcomes the level of HbA1c and FBS. The latter outcome as a clinical outcome is rather an unstable outcome measure. To have an accurate measure of clinical health state we need to have average values of HbA1c over at least 3 months. However, we had no access to such data from medical information systems. As a strategy to make our measurement complete, we used other clinical measures such as comorbidity and stage of diabetes.

## Conclusions

Considering several types of outcomes that measure the perception and evaluation of health services, we examined various aspects of patient experience with type 2 diabetes service delivery. By considering several operational factors we strived to guide interventions for improving health services delivery towards a patient-centred service delivery model. The operational factors of a patient-centred care model that significantly support the clinical outcomes and patient experience are as follows. Structural components comprise continuity, redistribution of tasks to low-cost resources, and improved access. Process components comprise a reduction in service utilization variation, increased responsiveness, more caring providers, more comprehensive care, and shared decision-making. Overall, the structure variables had a greater contribution to the outcomes. Based on these findings, interventions to improve outcomes firstly embark on variables of structure. In the next place, interventions may focus on disease-specific health behaviours, notably adherence to treatment recommendations.

## Supplementary Information

**Additional file 1 **Predictor variables, beta coefficient, confidence interval of coefficients, and *p*-value for statistical analyses of health outcomes.

## Data Availability

The datasets generated and analysed during the current study are not publicly available due to a lack of appropriate environment and approval for data sharing. Data that underlie our findings might be made available upon reasonable request. The lead investigator of this research will make data for this manuscript available upon request as possible in compliance with local research ethics board requirements and data sharing agreements.

## Abbreviations

T2D: Type 2 Diabetes
PCC: patient-centred care
MoHME: Iranian Ministry of Health and Medical Education
FBS: Fasting blood glucose
HbA1c: Glycated haemoglobin
