# Supplementary material for: Identifying associations between health services operational factors and health experience for patients with type 2 diabetes in Iran
Source: BMC Health Serv Res. 2021 Aug 31;21:896. doi: 10.1186/s12913-021-06932-0 (PMC8406836; doi:10.1186/s12913-021-06932-0)
Supplement: Supplementary file 1 — Additional file 1 Predictor variables, beta coefficient, confidence interval of coefficients, and p-value for statistical analyses of health outcomes. [file 12913_2021_6932_MOESM1_ESM.docx]

Table S 1 Predicting factors of HbA1c level; regression analysis of relationships between HbA1c level and demographic factors, socioeconomic factor, diabetes stage, general health behavior, disease-specific health behavior, diabetes care structure and processes among patients with type 2 diabetes

| Care model components | Variable | β | 95% Confidence interval | | P value |
| --- | --- | --- | --- | --- | --- |
| Demographic and socio-economic factors | Age | 0.01 | -0.02 | 0.04 | 0.377 |
|  | Sex |  |  |  |  |
|  | Female (reference) | 1 |  |  |  |
|  | Male | -0.26 | -0.86 | 0.35 | 0.401 |
|  | Education |  |  |  |  |
|  | Some years of schooling(reference) | 1 |  |  |  |
|  | High school diploma | 0.46 | -0.15 | 1.06 | 0.139 |
|  | University education | 0.42 | -0.49 | 1.34 | 0.363 |
| Diabetes stages | Disease state |  |  |  |  |
|  | Dependent on medication (reference) | 1 |  |  |  |
|  | Medication and/or insulin-dependent | 1.33 | 0.78 | 1.89 | P<.001 |
|  | Chronic comorbidity |  |  |  |  |
|  | Having no other chronic comorbidity (reference) | 1 |  |  |  |
|  | Having at least one other chronic comorbidity | -0.23 | -0.83 | 0.37 | 0.458 |
| General health behaviors | Physical exercises |  |  |  |  |
|  | Physical exercises <500 Metabolic Equivalents (METs) per week (reference) | 1 |  |  |  |
|  | Physical exercises >=500 Metabolic Equivalents (METs) per week | -0.07 | -1.01 | 0.88 | 0.891 |
|  | Smoking |  |  |  |  |
|  | Non-smoker (reference) | 1 |  |  |  |
|  | Former smoker | 0.23 | -1.24 | 1.71 | 0.755 |
|  | Current smoker | 1.58 | 0.62 | 2.55 | 0.001 |
| Diabetes-specific health behaviors/Self-care | Adherence to treatment (diet and medication and/or insulin injection) | -0.33 | -0.65 | -0.01 | 0.045 |
|  | Use of glucometer |  |  |  |  |
|  | Several times per day (reference) | 1 |  |  |  |
|  | Once per day | 1.23 | -0.13 | 2.58 | 0.076 |
|  | Once per some days | 1.46 | 0.39 | 2.52 | 0.008 |
|  | Once per some weeks | 1.12 | 0.04 | 2.19 | 0.042 |
|  | No use of glucometer | 1.20 | -0.01 | 2.41 | 0.052 |
| Diabetes care structures | Human resource model |  |  |  |  |
|  | Only family physician or general practitioner (reference) | 1 |  |  |  |
|  | Family physician or general practitioner & specialist physician | 0.92 | -0.43 | 2.27 | 0.179 |
|  | Only specialist physician | 0.50 | -0.73 | 1.73 | 0.426 |
|  | Access to diabetes services | -0.09 | -0.35 | 0.17 | 0.476 |
|  | Continuity of care |  |  |  |  |
|  | Being visited by a same doctor in every visit (reference) | 1 |  |  |  |
|  | Being visited by new doctor in every visit | 0.87 | 0.06 | 1.69 | 0.035 |
|  | Up to date equipment for diabetes care | 0.60 | 0.15 | 1.05 | 0.009 |
| Diabetes care processes | Comprehensiveness of medical consultation | -0.51 | -1.08 | 0.06 | 0.077 |
|  | Involvement in care decision by provider | 0.77 | 0.16 | 1.37 | 0.013 |
|  | Consistency of treatment medical plans and advices | -0.20 | -0.56 | 0.16 | 0.278 |
|  | Responsiveness of providers | -0.06 | -0.59 | 0.47 | 0.834 |
|  | Timeliness of provider | 0.08 | -0.53 | 0.7 | 0.786 |
|  | Caring provider | 0.27 | -0.47 | 1 | 0.472 |
|  | Politeness of care provider | -0.15 | -0.92 | 0.62 | 0.695 |
|  | Communication between patient and provider | -0.27 | -0.96 | 0.42 | 0.442 |

Table S 2 Predicting factors of Fasting Blood Glucose level; regression analysis of relationships between Fasting Blood Glucose level and demographic factors, socioeconomic factor, diabetes stage, general health behavior, disease-specific health behavior, diabetes care structure and processes among patients with type 2 diabetes

| Care model components | Variable | β | 95% Confidence interval | CI | P value |
| --- | --- | --- | --- | --- | --- |
| Demographic and socio-economic factors | Age | -0.24 | -0.95 | 0.47 | 0.51 |
|  | Sex |  |  |  |  |
|  | Female (reference) | 1 |  |  |  |
|  | Male | -10.61 | -25.37 | 4.16 | 0.16 |
|  | Education |  |  |  |  |
|  | Some years of schooling(reference) | 1 |  |  |  |
|  | High school diploma | -6.13 | -20.77 | 8.52 | 0.41 |
|  | University education | 8.19 | -15.86 | 32.23 | 0.50 |
| Diabetes stages | Disease state |  |  |  |  |
|  | Dependent on medication (reference) | 1 |  |  |  |
|  | Medication and/or insulin-dependent | 30.67 | 16.44 | 44.90 | 0 |
|  | Chronic comorbidity |  |  |  |  |
|  | Having no other chronic comorbidity (reference) | 1 |  |  |  |
|  | Having at least one other chronic comorbidity | 13.87 | 0.08 | 27.66 | 0.05 |
| General health behaviors | Physical exercises |  |  |  |  |
|  | Physical exercises <500 Metabolic Equivalents (METs) per week (reference) | 1 |  |  |  |
|  | Physical exercises >=500 Metabolic Equivalents (METs) per week | -17.71 | -34.56 | -0.85 | 0.04 |
|  | Smoking |  |  |  |  |
|  | Non-smoker (reference) | 1 |  |  |  |
|  | Former smoker | -0.29 | -28.37 | 27.80 | 0.98 |
|  | Current smoker | 13.14 | -13.02 | 39.30 | 0.32 |
| Diabetes-specific health behaviors/Self-care | Adherence to treatment (diet and medication and/or insulin injection) | -10.03 | -17.28 | -2.79 | 0.01 |
|  | Use of glucometer |  |  |  |  |
|  | Several times per day (reference) | 1 |  |  |  |
|  | Once per day | -7.29 | -43.00 | 28.42 | 0.69 |
|  | Once per some days | 16.34 | -12.37 | 45.06 | 0.26 |
|  | Once per some weeks | 9.94 | -19.47 | 39.36 | 0.51 |
|  | No use of glucometer | 7.40 | -23.42 | 38.22 | 0.64 |
| Diabetes care structures | Human resource model |  |  |  |  |
|  | Only family physician or general practitioner (reference) | 1 |  |  |  |
|  | Family physician or general practitioner & specialist physician | 12.21 | -9.77 | 34.19 | 0.28 |
|  | Only specialist physician | 3.18 | -16.06 | 22.42 | 0.74 |
|  | Access to diabetes services | -5.40 | -11.60 | 0.80 | 0.08 |
|  | Continuity of care |  |  |  |  |
|  | Being visited by a same doctor in every visit (reference) | 1 |  |  |  |
|  | Being visited by new doctor in every visit | 28.21 | 11.78 | 44.64 | 0.00 |
|  | Up to date equipment for diabetes care | 16.18 | 5.99 | 26.36 | 0.00 |
| Diabetes care processes | Comprehensiveness of medical consultation | -4.28 | -15.90 | 7.34 | 0.46 |
|  | Involvement in care decision by provider | 1.53 | -10.13 | 13.19 | 0.79 |
|  | Consistency of treatment medical plans and advices | 2.25 | -7.01 | 11.50 | 0.63 |
|  | Responsiveness of providers | -4.71 | -15.43 | 6.01 | 0.38 |
|  | Timeliness of provider | 7.59 | -5.26 | 20.44 | 0.24 |
|  | Caring provider | -16.67 | -30.76 | -2.59 | 0.02 |
|  | Politeness of care provider | 1.63 | -12.84 | 16.10 | 0.82 |
|  | Communication between patient and provider | 4.82 | -9.52 | 19.15 | 0.51 |

Table S 3 Predicting factors of EQ-5D quality of life; regression analysis of relationships between quality of life and demographic factors, socioeconomic factor, diabetes stage, general health behavior, disease-specific health behavior, diabetes care structure and processes among patients with type 2 diabetes

| Care model components | Variable | β | 95% Confidence interval | CI | P value |
| --- | --- | --- | --- | --- | --- |
| Demographic and socio-economic factors | Age | -0.00 | -0.01 | 0 | p<.001 |
|  | Sex |  |  |  |  |
|  | Female (reference) | 1 |  |  |  |
|  | Male | 0.08 | 0.04 | 0.12 | p<.001 |
|  | Education |  |  |  |  |
|  | Some years of schooling(reference) | 1 |  |  |  |
|  | High school diploma | -0.01 | -0.04 | 0.03 | 0.781 |
|  | University education | 0.00 | -0.06 | 0.06 | 0.972 |
| Diabetes stages | Disease state |  |  |  |  |
|  | Dependent on medication (reference) | 1 |  |  |  |
|  | Medication and/or insulin-dependent | -0.03 | -0.07 | 0 | 0.08 |
|  | Chronic comorbidity |  |  |  |  |
|  | Having no other chronic comorbidity (reference) | 1 |  |  |  |
|  | Having at least one other chronic comorbidity | -0.05 | -0.09 | -0.02 | 0.003 |
| General health behaviors | Physical exercises |  |  |  |  |
|  | Physical exercises <500 Metabolic Equivalents (METs) per week (reference) | 1 |  |  |  |
|  | Physical exercises >=500 Metabolic Equivalents (METs) per week | 0.02 | -0.02 | 0.06 | 0.395 |
|  | Smoking |  |  |  |  |
|  | Non-smoker (reference) | 1 |  |  |  |
|  | Former smoker | -0.09 | -0.16 | -0.02 | 0.012 |
|  | Current smoker | -0.05 | -0.12 | 0.01 | 0.122 |
| Diabetes-specific health behaviors/Self-care | Adherence to treatment (diet and medication and/or insulin injection) | 0.02 | 0.01 | 0.04 | 0.011 |
|  | Use of glucometer |  |  |  |  |
|  | Several times per day (reference) | 1 |  |  |  |
|  | Once per day | 0.02 | -0.07 | 0.11 | 0.626 |
|  | Once per some days | 0.03 | -0.05 | 0.1 | 0.493 |
|  | Once per some weeks | 0.02 | -0.06 | 0.09 | 0.619 |
|  | No use of glucometer | 0.03 | -0.05 | 0.11 | 0.425 |
| Diabetes care structures | Human resource model |  |  |  |  |
|  | Only family physician or general practitioner (reference) | 1 |  |  |  |
|  | Family physician or general practitioner & specialist physician | -0.01 | -0.06 | 0.05 | 0.794 |
|  | Only specialist physician | -0.02 | -0.07 | 0.02 | 0.316 |
|  | Access to diabetes services | 0.02 | 0 | 0.04 | 0.019 |
|  | Continuity of care |  |  |  |  |
|  | Being visited by a same doctor in every visit (reference) | 1 |  |  |  |
|  | Being visited by new doctor in every visit | -0.01 | -0.05 | 0.03 | 0.561 |
|  | Up to date equipment for diabetes care | -0.02 | -0.05 | 0 | 0.092 |
| Diabetes care processes | Comprehensiveness of medical consultation | 0.01 | -0.02 | 0.04 | 0.496 |
|  | Involvement in care decision by provider | 0 | -0.03 | 0.03 | 0.823 |
|  | Consistency of treatment medical plans and advices | 0.01 | -0.02 | 0.03 | 0.596 |
|  | Responsiveness of providers | 0 | -0.02 | 0.03 | 0.795 |
|  | Timeliness of provider | -0.01 | -0.04 | 0.02 | 0.607 |
|  | Caring provider | 0 | -0.04 | 0.04 | 0.986 |
|  | Politeness of care provider | 0.02 | -0.02 | 0.06 | 0.304 |
|  | Communication between patient and provider | -0.01 | -0.04 | 0.03 | 0.69 |

Table S 4 Predicting factors of visual analogue scale of EQ-5D; regression analysis of relationships between visual analogue scale of EQ-5D and demographic factors, socioeconomic factor, diabetes stage, general health behavior, disease-specific health behavior, diabetes care structure and processes among patients with type 2 diabetes

| Care model components | Variable | β | 95% Confidence interval | CI | P value |
| --- | --- | --- | --- | --- | --- |
| Demographic and socio-economic factors | Age | -0.13 | -0.38 | 0.11 | 0.28 |
|  | Sex |  |  |  |  |
|  | Female (reference) | 1 |  |  |  |
|  | Male | 5.53 | 0.49 | 10.57 | 0.03 |
|  | Education |  |  |  |  |
|  | Some years of schooling(reference) | 1 |  |  |  |
|  | High school diploma | 4.11 | -1.00 | 9.23 | 0.11 |
|  | University education | 6.50 | -1.70 | 14.70 | 0.12 |
| Diabetes stages | Disease state |  |  |  |  |
|  | Dependent on medication (reference) | 1 |  |  |  |
|  | Medication and/or insulin-dependent | -2.67 | -7.56 | 2.23 | 0.28 |
|  | Chronic comorbidity |  |  |  |  |
|  | Having no other chronic comorbidity (reference) | 1 |  |  |  |
|  | Having at least one other chronic comorbidity | -5.25 | -10.00 | -0.51 | 0.03 |
| General health behaviors | Physical exercises |  |  |  |  |
|  | Physical exercises <500 Metabolic Equivalents (METs) per week (reference) | 1 |  |  |  |
|  | Physical exercises >=500 Metabolic Equivalents (METs) per week | 11.33 | 5.59 | 17.07 | 0.00 |
|  | Smoking |  |  |  |  |
|  | Non-smoker (reference) | 1 |  |  |  |
|  | Former smoker | -0.33 | -9.94 | 9.28 | 0.95 |
|  | Current smoker | -4.28 | -13.23 | 4.67 | 0.35 |
| Diabetes-specific health behaviors/Self-care | Adherence to treatment (diet and medication and/or insulin injection) | 3.64 | 1.14 | 6.14 | 0.00 |
|  | Use of glucometer |  |  |  |  |
|  | Several times per day (reference) | 1 |  |  |  |
|  | Once per day | -4.10 | -16.49 | 8.29 | 0.52 |
|  | Once per some days | -7.93 | -18.02 | 2.17 | 0.12 |
|  | Once per some weeks | -4.18 | -14.50 | 6.13 | 0.43 |
|  | No use of glucometer | -10.72 | -21.44 | 0.00 | 0.05 |
| Diabetes care structures | Human resource model |  |  |  |  |
|  | Only family physician or general practitioner (reference) | 1 |  |  |  |
|  | Family physician or general practitioner & specialist physician | -0.23 | -7.72 | 7.26 | 0.95 |
|  | Only specialist physician | -2.76 | -9.34 | 3.82 | 0.41 |
|  | Access to diabetes services | 0.64 | -1.51 | 2.80 | 0.56 |
|  | Continuity of care |  |  |  |  |
|  | Being visited by a same doctor in every visit (reference) | 1 |  |  |  |
|  | Being visited by new doctor in every visit | -1.00 | -6.54 | 4.54 | 0.72 |
|  | Up to date equipment for diabetes care | -3.45 | -6.95 | 0.05 | 0.05 |
| Diabetes care processes | Comprehensiveness of medical consultation | 2.58 | -1.39 | 6.55 | 0.20 |
|  | Involvement in care decision by provider | 0.38 | -3.64 | 4.41 | 0.85 |
|  | Consistency of treatment medical plans and advices | 3.14 | 0.18 | 6.11 | 0.04 |
|  | Responsiveness of providers | 2.75 | -0.98 | 6.47 | 0.15 |
|  | Timeliness of provider | -1.45 | -5.92 | 3.03 | 0.53 |
|  | Caring provider | -1.57 | -6.47 | 3.33 | 0.53 |
|  | Politeness of care provider | 6.59 | 1.56 | 11.62 | 0.01 |
|  | Communication between patient and provider | -4.90 | -9.56 | -0.23 | 0.04 |

Table S 5 Predicting factors of satisfaction with health status; regression analysis of relationships between satisfaction with health status and demographic factors, socioeconomic factor, diabetes stage, general health behavior, disease-specific health behavior, diabetes care structure and processes among patients with type 2 diabetes

|  |  | β | 95% Confidence interval |  | P value |
| --- | --- | --- | --- | --- | --- |
| Demographic and socio-economic factors | Age | 0.23 | -0.03 | 0.50 | 0.09 |
|  | Sex |  |  |  |  |
|  | Female (reference) | 1 |  |  |  |
|  | Male | 5.64 | 0.12 | 11.16 | 0.05 |
|  | Education |  |  |  |  |
|  | Some years of schooling(reference) | 1 |  |  |  |
|  | High school diploma | 4.78 | -0.74 | 10.29 | 0.09 |
|  | University education | 1.86 | -7.11 | 10.83 | 0.68 |
| Diabetes stages | Disease state |  |  |  |  |
|  | Dependent on medication (reference) | 1 |  |  |  |
|  | Medication and/or insulin-dependent | -3.01 | -8.37 | 2.34 | 0.27 |
|  | Chronic comorbidity |  |  |  |  |
|  | Having no other chronic comorbidity (reference) |  |  |  |  |
|  | Having at least one other chronic comorbidity | -8.43 | -13.62 | -3.23 | 0.00 |
| General health behaviors | Physical exercises |  |  |  |  |
|  | Physical exercises <500 Metabolic Equivalents (METs) per week (reference) | 1 |  |  |  |
|  | Physical exercises >=500 Metabolic Equivalents (METs) per week | 7.23 | 0.96 | 13.50 | 0.02 |
|  | Smoking |  |  |  |  |
|  | Non-smoker (reference) | 1 |  |  |  |
|  | Former smoker | 2.35 | -8.16 | 12.85 | 0.66 |
|  | Current smoker | -3.09 | -12.89 | 6.71 | 0.54 |
| Diabetes-specific health behaviors/Self-care | Adherence to treatment (diet and medication and/or insulin injection) | 3.63 | 0.91 | 6.35 | 0.01 |
|  | Use of glucometer |  |  |  |  |
|  | Several times per day (reference) | 1 |  |  |  |
|  | Once per day | -8.58 | -22.04 | 4.88 | 0.21 |
|  | Once per some days | -8.46 | -19.23 | 2.30 | 0.12 |
|  | Once per some weeks | -3.28 | -14.29 | 7.73 | 0.56 |
|  | No use of glucometer | -10.87 | -22.34 | 0.60 | 0.06 |
| Diabetes care structures | Human resource model |  |  |  |  |
|  | Only family physician or general practitioner (reference) | 1 |  |  |  |
|  | Family physician or general practitioner & specialist physician | -8.05 | -16.27 | 0.17 | 0.06 |
|  | Only specialist physician | -9.03 | -16.23 | -1.83 | 0.01 |
|  | Access to diabetes services | 1.42 | -0.93 | 3.77 | 0.24 |
|  | Continuity of care |  |  |  |  |
|  | Being visited by a same doctor in every visit (reference) | 1 |  |  |  |
|  | Being visited by new doctor in every visit | 0.14 | -5.91 | 6.19 | 0.96 |
|  | Up to date equipment for diabetes care | -2.72 | -6.55 | 1.11 | 0.16 |
| Diabetes care processes | Comprehensiveness of medical consultation | 1.88 | -2.47 | 6.24 | 0.40 |
|  | Involvement in care decision by provider | -1.62 | -5.99 | 2.75 | 0.47 |
|  | Consistency of treatment medical plans and advices | 1.90 | -1.34 | 5.14 | 0.25 |
|  | Responsiveness of providers | 3.89 | -0.19 | 7.96 | 0.06 |
|  | Timeliness of provider | -0.56 | -5.42 | 4.30 | 0.82 |
|  | Caring provider | 0.28 | -5.06 | 5.63 | 0.92 |
|  | Politeness of care provider | 0.43 | -4.97 | 5.82 | 0.88 |
|  | Communication between patient and provider | -1.01 | -6.10 | 4.08 | 0.70 |

Table S 6 Predicting factors of satisfaction with diabetes services; regression analysis of relationships between satisfaction with diabetes services and demographic factors, socioeconomic factor, diabetes stage, general health behavior, disease-specific health behavior, diabetes care structure and processes among patients with type 2 diabetes

| Care model components | Variable | β | 95% Confidence interval | | P value |
| --- | --- | --- | --- | --- | --- |
| Demographic and socio-economic factors | Age | -0.19 | -0.43 | 0.06 | 1.36 |
|  | Sex |  |  |  |  |
|  | Female (reference) | 1 |  |  |  |
|  | Male | 1.06 | -4.01 | 6.12 | 0.68 |
|  | Education |  |  |  |  |
|  | Some years of schooling(reference) | 1 |  |  | 0.24 |
|  | High school diploma | -3.07 | -8.19 | 2.05 | 0.44 |
|  | University education | -3.24 | -11.44 | 4.97 |  |
| Diabetes stages | Disease state |  |  |  |  |
|  | Dependent on medication (reference) | 1 |  |  |  |
|  | Medication and/or insulin-dependent | -1.83 | -6.76 | 3.10 | 0.46 |
|  | Chronic comorbidity |  |  |  |  |
|  | Having no other chronic comorbidity (reference) | 1 |  |  |  |
|  | Having at least one other chronic comorbidity | 0.37 | -4.36 | 5.10 | 0.88 |
| General health behaviors | Physical exercises |  |  |  |  |
|  | Physical exercises <500 Metabolic Equivalents (METs) per week (reference) | 1 |  |  |  |
|  | Physical exercises >=500 Metabolic Equivalents (METs) per week | -0.04 | -5.77 | 5.70 | 0.99 |
|  | Smoking |  |  |  |  |
|  | Non-smoker (reference) | 1 |  |  |  |
|  | Former smoker | -0.61 | -10.17 | 8.96 | 0.91 |
|  | Current smoker | -8.98 | -17.93 | -0.02 | 0.05 |
| Diabetes-specific health behaviors/Self-care | Adherence to treatment (diet and medication and/or insulin injection) | 3.31 | 0.84 | 5.78 | 0.01 |
|  | Use of glucometer |  |  |  |  |
|  | Several times per day (reference) | 1 |  |  |  |
|  | Once per day | 1.78 | -10.38 | 13.94 | 0.77 |
|  | Once per some days | 4.34 | -5.51 | 14.18 | 0.38 |
|  | Once per some weeks | 3.92 | -6.15 | 14.00 | 0.44 |
|  | No use of glucometer | 3.33 | -7.11 | 13.77 | 0.53 |
| Diabetes care structures | Human resource model |  |  |  |  |
|  | Only family physician or general practitioner (reference) | 1 |  |  |  |
|  | Family physician or general practitioner & specialist physician | 5.59 | -1.86 | 13.04 | 0.14 |
|  | Only specialist physician | 3.54 | -3.01 | 10.09 | 0.29 |
|  | Access to diabetes services | 5.49 | 3.32 | 7.66 | 0.00 |
|  | Continuity of care |  |  |  |  |
|  | Being visited by a same doctor in every visit (reference) | 1 |  |  |  |
|  | Being visited by new doctor in every visit | 0.19 | -5.32 | 5.69 | 0.94 |
|  | Up to date equipment for diabetes care | 3.18 | -0.33 | 6.69 | 0.08 |
| Diabetes care processes | Comprehensiveness of medical consultation | 4.83 | 0.74 | 8.91 | 0.02 |
|  | Involvement in care decision by provider | -2.13 | -6.35 | 2.09 | 0.32 |
|  | Consistency of treatment medical plans and advices | -1.08 | -4.15 | 1.99 | 0.48 |
|  | Responsiveness of providers | 3.96 | 0.19 | 7.74 | 0.04 |
|  | Timeliness of provider | -1.38 | -5.78 | 3.02 | 0.54 |
|  | Caring provider | 6.62 | 1.74 | 11.49 | 0.01 |
|  | Politeness of care provider | 1.00 | -3.89 | 5.88 | 0.69 |
|  | Communication between patient and provider | -2.72 | -7.42 | 1.98 | 0.26 |

Table S 7 Predicting factors of the evaluation of health services in comparison with best and worst imaginable diabetes services; regression analysis of relationships between the evaluation of health services in comparison with best and worst imaginable diabetes services and demographic factors, socioeconomic factor, diabetes stage, general health behavior, disease-specific health behavior, diabetes care structure and processes among patients with type 2 diabetes

| Care model components | Variable | β | 95% Confidence interval | CI | P value |
| --- | --- | --- | --- | --- | --- |
| Demographic and socio-economic factors | Age | 0.06 | -0.19 | 0.31 | 0.64 |
|  | Sex |  |  |  |  |
|  | Female (reference) | 1 |  |  |  |
|  | Male | 1.64 | -3.51 | 6.80 | 0.53 |
|  | Education |  |  |  |  |
|  | Some years of schooling(reference) | 1 |  |  |  |
|  | High school diploma | -0.50 | -5.65 | 4.65 | 0.85 |
|  | University education | -1.66 | -9.97 | 6.65 | 0.70 |
| Diabetes stages | Disease state |  |  |  |  |
|  | Dependent on medication (reference) | 1 |  |  |  |
|  | Medication and/or insulin-dependent | -0.25 | -5.24 | 4.73 | 0.92 |
|  | Chronic comorbidity |  |  |  |  |
|  | Having no other chronic comorbidity (reference) | 1 |  |  |  |
|  | Having at least one other chronic comorbidity | -1.06 | -5.90 | 3.78 | 0.67 |
| General health behaviors | Physical exercises |  |  |  |  |
|  | Physical exercises <500 Metabolic Equivalents (METs) per week (reference) | 1 |  |  |  |
|  | Physical exercises >=500 Metabolic Equivalents (METs) per week | -1.38 | -7.15 | 4.39 | 0.64 |
|  | Smoking |  |  |  |  |
|  | Non-smoker (reference) | 1 |  |  |  |
|  | Former smoker | 5.31 | -4.37 | 14.99 | 0.28 |
|  | Current smoker | -6.04 | -15.06 | 2.99 | 0.19 |
| Diabetes-specific health behaviors/Self-care | Adherence to treatment (diet and medication and/or insulin injection) | 2.81 | 0.31 | 5.31 | 0.03 |
|  | Use of glucometer |  |  |  |  |
|  | Several times per day (reference) | 1 |  |  |  |
|  | Once per day | 5.25 | -7.81 | 18.32 | 0.43 |
|  | Once per some days | 4.34 | -5.83 | 14.52 | 0.40 |
|  | Once per some weeks | 6.10 | -4.29 | 16.50 | 0.25 |
|  | No use of glucometer | 4.20 | -6.59 | 15.00 | 0.44 |
| Diabetes care structures | Human resource model |  |  |  |  |
|  | Only family physician or general practitioner (reference) | 1 |  |  |  |
|  | Family physician or general practitioner & specialist physician | 2.62 | -4.92 | 10.16 | 0.49 |
|  | Only specialist physician | 3.28 | -3.35 | 9.91 | 0.33 |
|  | Access to diabetes services | 1.65 | -0.52 | 3.81 | 0.14 |
|  | Continuity of care |  |  |  |  |
|  | Being visited by a same doctor in every visit (reference) | 1 |  |  |  |
|  | Being visited by new doctor in every visit | -3.73 | -9.31 | 1.86 | 0.19 |
|  | Up to date equipment for diabetes care | 3.02 | -0.51 | 6.55 | 0.09 |
| Diabetes care processes | Comprehensiveness of medical consultation | 1.95 | -2.13 | 6.03 | 0.35 |
|  | Involvement in care decision by provider | 2.27 | -1.78 | 6.31 | 0.27 |
|  | Consistency of treatment medical plans and advices | 1.17 | -1.82 | 4.15 | 0.44 |
|  | Responsiveness of providers | 3.94 | 0.19 | 7.69 | 0.04 |
|  | Timeliness of provider | 1.53 | -2.91 | 5.97 | 0.50 |
|  | Caring provider | 6.59 | 1.68 | 11.49 | 0.01 |
|  | Politeness of care provider | 0.44 | -4.49 | 5.37 | 0.86 |
|  | Communication between patient and provider | -3.80 | -8.49 | 0.88 | 0.11 |
